# EmbA is an essential arabinosyltransferase in *Mycobacterium tuberculosis*

**DOI:** 10.1099/mic.0.2007/012153-0

**Published:** 2008-01

**Authors:** Anita G. Amin, Renan Goude, Libin Shi, Jian Zhang, Delphi Chatterjee, Tanya Parish

**Affiliations:** 1 Department of Microbiology, Immunology and Pathology, Colorado State University, CO 80523, USA; 2 Centre for Infectious Disease, Barts and the London, Queen Mary's School of Medicine and Dentistry, London E1 2AT, UK

## Abstract

The Emb proteins (EmbA, EmbB, EmbC) are mycobacterial arabinosyltransferases involved in the biogenesis of the mycobacterial cell wall. EmbA and EmbB are predicted to work in unison as a heterodimer. EmbA and EmbB are involved in the formation of the crucial terminal hexaarabinoside motif [Ara*β*(1→2)Ara*α*(1→5)] [Ara*β*(1→2)Ara*α*(1→3)]Ara*α*(1→5)Ara*α*1→(Ara_6_) in the cell wall polysaccharide arabinogalactan. Studies conducted in *Mycobacterium smegmatis* revealed that mutants with disruptions in *embA* or *embB* are viable, although the growth rate was affected. In contrast, we demonstrate here that *embA* is an essential gene in *Mycobacterium tuberculosis*, since a deletion of the chromosomal gene could only be achieved when a second functional copy was provided on an integrated vector. Complementation of an *embA* mutant of *M. smegmatis* by *M. tuberculosis embA* confirmed that it encodes a functional arabinosyltransferase. We identified a promoter for *M. tuberculosis embA* located immediately upstream of the gene, indicating that it is expressed independently from the upstream gene, *embC*. Promoter activity from P*_embA_*_(Mtb)_ was sevenfold lower when assayed in *M. smegmatis* compared to *M. tuberculosis*, indicating that the latter is not a good host for genetic analysis of *M. tuberculosis embA* expression. P*_embA_*_(Mtb)_ activity remained constant throughout growth phases and after stress treatment, although it was reduced during hypoxia-induced non-replicating persistence. Ethambutol exposure had no effect on P*_embA_*_(Mtb)_ activity. These data demonstrate that *M. tuberculosis* *embA* encodes a functional arabinosyltransferase which is constitutively expressed and plays a critical role in *M. tuberculosis*.

## INTRODUCTION

*Mycobacterium tuberculosis* poses a serious global health problem, causing millions of deaths and new infections every year. There is an urgent need for new antimycobacterial therapeutics, since the current treatment regimen is lengthy and ineffective against multi-drug-resistant strains. The cell wall of *M. tuberculosis* is an attractive drug target, since it is critical for cell survival and a number of current antitubercular agents target this structure. In mycobacteria, the peptidoglycan, located immediately outside the cytoplasmic membrane, is covalently attached to arabinogalactan (AG), which is in turn esterified to the mycolic acid layer. This structure, called the mycolyl-AG-peptidoglycan (mAGP) complex, forms the core framework of the mycobacterial cell wall. The wall provides a hydrophobic permeability barrier and is responsible for at least part of the intrinsic resistance of mycobacteria to a number of antibiotics (Brennan, 2003).

Arabinosyltransferases, encoded by *emb* genes, play key roles in the synthesis of mycobacterial cell wall components. Two *emb* genes (*embA* and *embB*) were initially identified in *Mycobacterium avium* (Belanger *et al.*, 1996). Subsequently, three Emb proteins have been identified in *M. tuberculosis* (Cole *et al.*, 1998) and in *Mycobacterium smegmatis* (Escuyer *et al.*, 2001); no orthologues have been identified elsewhere than in the order *Actinomycetales*. The genomic arrangement of the *emb* genes in *M. tuberculosis* and *M. smegmatis* is similar, with the three genes (*embCAB*) being co-localized and possibly being transcribed as an operon (Telenti *et al.*, 1997).

The Emb proteins form a family of large (>1100 residues) transmembrane proteins with a cytoplasmic N-terminal domain, 13–15 transmembrane segments and a large extracytoplasmic C-terminal domain (Berg *et al.*, 2003; Seidel *et al.*, 2007). Emb proteins are considered to be good drug targets, since the current antitubercular drug, ethambutol, causes cessation of the synthesis of arabinan polymers [AG and lipoarabinomannan (LAM)]. This is due to the inhibition of arabinosyltransferases (Khoo *et al.*, 1996; Mikusova *et al.*, 1995) and *embB* mutations have been associated with ethambutol resistance in *M. tuberculosis* (Sreevatsan *et al.*, 1997).

Almost all of the work characterizing the role and expression of Emb proteins has been carried out in the saprophytic species *M. smegmatis* where the role of each protein has been determined. Biochemical analyses of deletion mutants has clearly shown that the Emb proteins are all arabinosyltransferases, but with segregated biological functions. EmbC is involved in the biosynthesis of the arabinan portion of LAM (Zhang *et al.*, 2003), whereas EmbA and EmbB are involved in AG synthesis (Escuyer *et al.*, 2001). Thus, the crucial hexaarabinoside terminal motif, which is the structural motif for mycolylation in AG (McNeil *et al.*, 1994), is altered in both *M. smegmatis embA* and *embB* mutants (Escuyer *et al.*, 2001). An EmbA deletion mutant has a number of other phenotypic changes, including altered morphology, slight loss of acid-fastness and increased susceptibility to hydrophobic antibiotics (Escuyer *et al.*, 2001). These changes are likely to be a direct result of the defective synthesis of AG in the cell wall.

No direct information on the role of *M. tuberculosis* EmbA (EmbA_Mtb_) has been obtained to date and there is no information on the expression of the *embA* gene or its promoter in this species. To determine the function of EmbA_Mtb_, we attempted to construct a deletion mutant by gene replacement. We demonstrate here that *embA* is essential in *M. tuberculosis* under normal culture conditions. EmbA_Mtb_ was confirmed as a bona fide arabinosyltransferase, since it was able to complement an *M. smegmatis embA* mutant in an *in vitro* assay. We also identified the promoter of *embA* and demonstrated that its expression is constitutive.

## METHODS

### Culture.

Mycobacteria were grown in Middlebrook liquid medium (7H9-OADC) [4.7 g Middlebrook 7H9 l^−1^ plus 10 % (v/v) OADC (oleic acid, albumin, dextrose, catalase) supplement (Becton Dickinson)] with 0.05 % (w/v) Tween 80 (Tw) where stated, or on Middlebrook solid medium (7H10-OADC) [19 g Middlebrook 7H10 l^−1^ plus 10 % (v/v) OADC supplement]. Dubos medium supplemented with 5 % (w/v) glycerol and 10 % (v/v) Dubos medium albumin (Becton Dickinson) was used for hypoxic cultures. Aerobic liquid cultures of *M. tuberculosis* were static 10 ml cultures, inoculated 1 : 10 in 50 ml tubes. Hypoxic cultures were performed in 17 ml medium in 20 mm glass tubes with slow stirring (50 r.p.m.) from a starting OD_570_ of 0.004. Kanamycin was used at 20 μg ml^−1^, hygromycin at 100 μg ml^−1^, streptomycin at 20 μg ml^−1^, gentamicin at 10 μg ml^−1^, X-Gal at 50 μg ml^−1^ and sucrose at 5 % (w/v) unless otherwise indicated.

### Construction of *embA* deletion vector.

A deletion delivery vector for *embA* was constructed as follows: the upstream and downstream regions of *embA* were amplified using primer pairs F1 (5′-ATCGCAGTTTCCTCAACGAC-3′) and R1 (5′-CCTCGAGGGATCGAGATGTCCAG-3′), and F2 (5′-CTCGAGGTCGTCGAACCTATGGCAGT-3′) and R2 (5′-AGCGCCAGCAGGTTGTAATA-3′), respectively, and cloned into pGEM-T Easy (Promega). *Xho*I restriction sites (underlined) were engineered into the primers. The two fragments were excised as *Kpn*I–*Xho*I and *Xho*I–*Bam*HI fragments and cloned into p2NIL (Parish & Stoker, 2000). The *lacZ*, *sacB*, *hyg* cassette from the marker cassette vector pGOAL19 (Parish & Stoker, 2000) was excised as a *Pac*I fragment and inserted into p2NIL to make the final vector, pEMPTY16.

### Attempts to construct an *embA* deletion strain.

Plasmid pEMPTY16 was pre-treated with UV to promote homologous recombination and electroporated into *M. tuberculosis* (Hinds *et al.*, 1999). Single cross-overs (SCOs) were isolated on hygromycin, kanamycin and X-Gal plates. One SCO was streaked onto solid medium without antibiotics to allow the second cross-over to occur. Double cross-overs (DCOs) were isolated on sucrose, X-Gal plates. White colonies were patch-tested for kanamycin and hygromycin sensitivity to confirm vector loss. A PCR screen using primers DIET1 (5′-CATCCTCACCGCCCTTAAC-3′) and DIET2 (5′-CGATTTGGGGTGCTTTTG-3′) was used to distinguish between wild-type (3.4 kb band) and deletion DCOs (0.5 kb band).

### Construction of *embA* merodiploid strain.

The complementation vector pEMPTY22 was constructed by amplifying the *embA* gene with primers Empathy3 (5′-TTAATTAATGGCCAGCTACCTCAAAGAC-3′) and Empathy4 (5′-TTAATTAAACCGACAACACAAAGCCAAT-3′), cloning into pGEM-T Easy and subcloning into pAPA3 (L5 integrating vector with Ag85a promoter; unpublished) as a *Pac*I fragment (sites underlined) in the correct orientation for expression from the Ag85a promoter. pEMPTY22 was transformed into the SCO strain to generate a merodiploid strain (Tame 68). DCOs were isolated from this strain by plating onto sucrose, X-Gal and gentamicin. Sucrose-resistant, white colonies were screened by PCR as before. ‘Del-int ’ DCO strains (one deleted copy and one integrated copy of *embA*) were isolated and confirmed by Southern analysis. For gene switching, two del-int strains [*embA*Δ (P_Ag85a_-*embA*, L5 *int*, *Gm*)] were transformed with the integrating vector pUC-Hyg-Int (Mahenthiralingam *et al.*, 1998). Transformants were plated on hygromycin±gentamicin and patch-tested for hygromycin/gentamicin resistance.

### Analyses of promoter activity.

The intergenic region between *embC* and *embA* of *M. tuberculosis* was amplified using primers 5′-CCCAGTACTAGCGGTTGACGCCTTACTAC-3′ and 5′-CCCAGTACTAGATCGCTCATTACCGTCGT-3′, cloned as a *Sca*I fragment (sites underlined) into the L5-based integrating vector pSM128 (Dussurget *et al.*, 1999) upstream of the promoterless *lacZ* gene to make plasmid pEMBA and the sequence verified. For site-directed mutagenesis, primers SDM1 (5′-CGC GTC GCC GAC CAG CGA GCC TCG-3′) and SDM2 (5′-CGA GGC TCG CTG GTC GGC GAC GCG-3′) (mutated nucleotides underlined) were used to generate a double mutation. The amplification reaction for site-directed mutagenesis was carried out in 50 μl total volume containing 1× *Pfu* Ultra reaction buffer, 0.5 mM dNTPs, 10 pmol each primer, 10 % DMSO, 10 ng template and 2.5 units *Pfu* Ultra (Stratagene). The thermocycling programme used was 95 °C for 1 min, followed by 18 cycles of 95 °C for 1 min, 60 °C for 1 min, 68 °C for 12 min and a final extension cycle at 68 °C for 20 min. The template was degraded using 10 units *Dpn*I at 37 °C for 1 h. Five microlitres of the reaction was then used to transform competent *Escherichia coli*. Recombinant pEMBA-M plasmid was isolated and the sequence verified. Plasmids pEMBA and pEMBA-M were electroporated into *M. smegmatis* and *M. tuberculosis* and streptomycin-resistant transformants were isolated. Three independent transformants for each were selected for promoter activity determinations. Cell-free extracts were prepared (Parish & Wheeler, 1998) and *β*-galactosidase assays were performed as described by Miller (1972).

### Isolation of RNA and identification of the *embA* transcript.

Total RNA was isolated from *M. tuberculosis* cells grown in 100 ml 7H9-OADC containing 0.05 % (w/v) Tween 80 to an OD_600_ of 0.8. The cells were pelleted at 3000 ***g*** for 10 min and resuspended in 10 ml Trizol (Invitrogen), vortexed and frozen at –80 °C for 1 h. The cells were broken in a FastPrep machine (Thermo Electron) at speed 6.0 for 40 s and the lysate was extracted with 2 ml chloroform. The cell lysate was centrifuged at 27 000 ***g*** at 4 °C for 30 min and the aqueous layer was precipitated with 5 ml 2-propanol at −80 °C overnight, the pellet being collected by centrifugation at 14 000 ***g*** for 10 min at 4 °C, washed with 70 % ethanol, dried and dissolved in 100 μl RNase-free water. The sample was then treated with 5 units RNase-free DNase for 1 h at 37 °C. After the removal of DNase by phenol/chloroform extraction, the RNA was precipitated with ethanol and dissolved in 50 μl deionized RNase-free water. cDNA was generated using Superscript III reverse transcriptase (RT) (Invitrogen) with the primers EMBAR (5′-CGATGGCCGAGCAGGGGATC-3′) and EMBBR (5′-GGTCGGTGACGTCCACGCG-3′). PCR was performed using primer pairs EMBAR and EMBCF2 (5′-GCGAGGTCCGGTTGCAGTGG-3′), and EMBBR and EMBAF1 (5′-CTGCCAGCGACCGTTTTCC-3′) in a 50 μl reaction using *Taq* DNA polymerase and 2 μl cDNA from the first strand synthesis. The amplification was performed with an initial denaturation at 94 °C for 5 min, followed by 30 cycles of denaturation at 94 °C for 30 s, annealing at 55 °C for 30 s, and extension at 72 °C for 1 min, followed by a final extension step at 72 °C for 7 min.

For Northern analysis, 16 μg total RNA from *M. tuberculosis* was separated by gel electrophoresis using 1 % agarose gel and transferred to a positively charged nylon membrane (Amersham Hybond-N+) for 1.5 h using the QBIOgene vacuum blotter. The PCR-amplified *embA*_Mtb_ was purified and used as the hybridization probe. Labelling and detection were carried out using the AlkPhos Direct kit (Amersham), according to the manufacturer's instructions.

### Arabinosyltransferase assay.

The 3285 bp *embA*_Mtb_ was amplified from genomic DNA using the primers TAF1 (5′-CCCCATATGGTGCCCCACGACGGTAAT-3′) and TAR1 (5′-CCCAAGCTTTCATGGCAGCGCCCTGAT-3′) and the 3279 bp *embA* gene from *M. smegmatis* (*embA*_Msm_) was amplified from genomic DNA using the primers TMF1 (5′-CCCCATATGGTGCCGGGCGATGAACAG-3′) and TMR1 (5′-CCCAAGCTTTCACGGCAGTGCCCTGAT-3′) with both primer sets having *Nde*I and *Hin*dIII restriction sites (underlined) in the forward and reverse primers, respectively. The amplified genes were cloned into the pVV16 expression vector to make pTA and pMA, respectively, and the sequences were verified. Plasmids pTA and pMA were transformed into an *embA*_Msm_ knockout strain (Δ*embA*_Msm_) (Escuyer *et al.*, 2001) to generate Δ*embA*_Msm_, complemented with *embA*_Mtb_, and Δ*embA*_Msm_, complemented with *embA*_Msm_, respectively.

For the arabinosyltransferase assay, strains were grown to an OD_600_ of 0.8. Cell wall (P60) and membrane fractions were purified from the strains by standard procedures (Khasnobis *et al.*, 2006). The arabinosyltransferase assay reaction mixture contained buffer A (50 mM MOPS, pH 8.0, 5 mM *β*-mercaptoethanol, 10 mM MgCl_2_), 1 mM ATP, 500 000 d.p.m. p[^14^C]Rpp (^14^C-labelled phosphoribosylpyrophosphate, used as a donor to form decaprenylphosphoryl-d-[^14^C]arabinose *in situ*) (Scherman *et al.*, 1996), 300 μM acceptor [pentasaccharide, octyl-*β*-d-Ara*f*-(1→2)-*α*-d-Ara*f*-(1→5)-*α*-d-Ara*f*-(1→5)-*α*-d-Ara*f*-(1→5)-*α*-d-Ara*f*, used as a substrate for arabinosyltransferase activity in a cell-free assay] (Khasnobis *et al.*, 2006), 0.3 mg P60 and 0.8 mg membrane fractions in a total volume of 200 μl. Reactions were incubated at 37 °C for 1 h, terminated by adding 200 μl ethanol and centrifuged briefly at 14 000 ***g***, and the supernatant was loaded onto a pre-packed strong anion exchange (SAX) column (Burdick and Jackson) pre-equilibrated with 5 ml water. Columns were eluted with 2 ml water, and the eluants were dried and partitioned between the two phases (1 : 1) of water-saturated 1-butanol and water, three times; the 1-butanol fractions collected were air-dried, reconstituted in 200 μl 1-butanol and measured by liquid scintillation spectrometry. 3000 d.p.m. were analysed by silica gel TLC developed in CHCl_3_/CH_3_OH/1 M NH_4_OAc/NH_4_OH/H_2_O (180 : 140 : 9 : 9 : 23), exposed for 5 days and visualized using a phosphoimager (Molecular Dynamics). The product was purified from the TLC, and approximately 5000 d.p.m. of the product was treated with endoarabinanase isolated from *Cellulomonas gelida* (McNeil *et al.*, 1994) for 16 h at 37 °C (Khoo *et al.*, 1996) and subjected to Dionex high-pH anion-exchange chromatography (HPAEC).

### Preparation of LAM and soluble AG.

Cells (10 g wet wt) were delipidated with hot absolute ethanol followed by a 2 : 1 chloroform/methanol extraction (Khoo *et al.*, 1996). Delipidated cells were resuspended in PBS and sonicated using Soniprep 150 (Sanyo, MSE Ltd). The resulting suspension was extracted three times with 50 % ethanol and the 27 000 ***g*** supernatants were combined, concentrated and digested with 1 mg proteinase K ml^−1^. After dialysis, aliquots of the aqueous solution containing LAM, lipomannan and phosphatidyl-*myo*-inositol mannosides were examined by SDS-PAGE. The remainder was applied to a Sephacryl S-200 (Pharmacia) column as described by Khoo *et al.* (1996). SDS-PAGE was used to monitor the elution profile, and fractions that contained LAM were individually pooled and dialysed. The cell-wall core mAGP complex was obtained from the residual pellet after LAM, lipomannan and phosphatidyl-*myo*-inositol mannoside extraction as described by Escuyer *et al.* (2001). To release soluble AG from peptidoglycans, the insoluble residue (mAGP complex) was treated with 2 M NaOH for 16 h at 80 °C and the soluble AG was purified as described by Daffe *et al.* (1990).

### GC and GC/MS analysis of glycosyl composition.

Alditol acetates for GC were prepared as described by McNeil *et al.* (1989). GC of the alditol acetates was performed on an HP Gas Chromatograph Model 5890 fitted with an SP 2380 (30 m×0.25 mm i.d.; Supelco) column at an initial temperature of 50 °C, held for 1 min. The temperature was raised to 170 °C at 30 °C min^−1^ before increasing to 260 °C at 5 °C min^−1^. For GC/MS, partially methylated alditol acetates were dissolved in chloroform prior to injection on a DB-5 column (10 m×0.18 mm i.d.; J&W Scientific) using a ThermoQuest Trace Gas Chromatograph 2000 (ThermoQuest) connected to a GCQ/Polaris MS mass detector at an initial temperature of 50 °C held for 1 min. The temperature was raised to 150 °C at 30 °C min^−1^ before increasing to 260 °C at 5 °C min^−1^.

## RESULTS

### *embA* is an essential gene in *M. tuberculosis*

**Fig. 1. f1:**
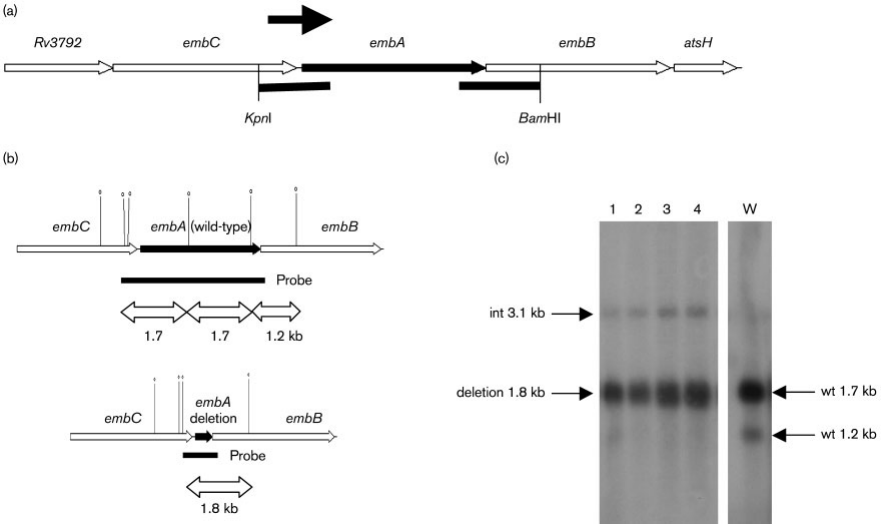
Demonstration of essentiality of *embA* in *M. tuberculosis*. (a) The genomic organization of the *embA* region is shown. The regions marked by black bars were amplified and cloned into p2NIL to generate the delivery vector pEMPTY16. The promoter region assayed for activity is indicated by an arrow. (b) and (c) Southern analysis of recombinant strains. (b) The maps of the wild-type and deletion mutant are shown, with the expected sizes. *Bam*HI sites are indicated by symbols (0) and the probe is shown as a solid bar. (c) No deletion DCOs were isolated in the wild-type background. PCR was used to screen for potential deletion alleles in the merodiploid background and four potential del-int strains were identified. Genomic DNA from these four strains was digested with *Bam*HI and hybridized to the probe. The expected sizes for the wild-type and integrated (int) copies of *embA* are given. Lanes: 1–4, genomic DNA from four del-int strains (deletion DCOs with integrated *embA*); W, wild-type genomic DNA.

We were interested in exploring the role of EmbA in *M. tuberculosis*. Although data were available demonstrating its function in *M. smegmatis* (Escuyer *et al.*, 2001), nothing was known of its function in *M. tuberculosis*. We first attempted to make an *embA* deletion in *M. tuberculosis* using a two-step recombination method (Parish & Stoker, 2000). The deletion vector carried an unmarked, in-frame deletion of the majority of the *embA* gene to ensure that any mutations did not affect expression of the downstream genes (Fig. 1a[Fig f1]). We screened 40 DCO strains and all were wild-type. This was unexpected, since gene knockouts of *embA* were readily obtained in *M. smegmatis* (Escuyer *et al.*, 2001). To confirm essentiality, we constructed a merodiploid strain carrying a second functional copy of *embA* under the control of the constitutive Ag85a promoter in an L5 integrating vector. In this background we were able to isolate DCOs with both the deletion and the wild-type allele (Fig. 1). Four out of 24 DCOs in the merodiploid background were deletions, showing that *embA* is an essential gene in *M. tuberculosis* (Fisher's exact *t*-test, *P*=0.02). The genotype of the DCO strains was confirmed by Southern blotting (Fig. 1c[Fig f1]).

### Gene switching

Since the essentiality of *embA* was not expected, we confirmed our results using a second method (Pashley & Parish, 2003). We showed that we could not replace the integrated vector pEMPTY22 (*embA*, *L5 int*, *Gm*) when it carried the only functional copy of *embA*. Two del-int strains [Tame 91 and Tame 93: *embA*Δ (*embA*, *L5 int*, *Gm*)] were transformed with an empty integrating vector pUC-Hyg-Int (*L5 int*, *hyg*) and transformants were selected on hygromycin. If the gene was non-essential, the incoming vector would replace the resident integrated vector at a high frequency and hygromycin-resistant (gentamicin-sensitive) colonies would be isolated. If the gene was essential, then only co-integrated strains (pEMPTY22 and pUC-Hyg-Int) would be recovered; this occurs at a low frequency and strains are gentamicin- and hygromycin-resistant. We carried out this gene switching experiment with two independent del-int strains (Tame 91 and Tame 93) (Table 1). The results indicated that replacement of pEMPTY22 did not occur. The numbers of hygromycin-resistant transformants were 10^2^- to 10^3^-fold lower with the switching vector than with the control vector. In both cases, 24 hygromycin-resistant colonies were patch-tested and all were gentamicin-resistant, confirming that the original plasmid containing *embA* was still present. Since the resident vector could not be replaced, this further confirms that *embA* is an essential gene in *M. tuberculosis*.

### *embA*_Mtb_ encodes an arabinosyltransferase

*embA*_Mtb_ has been proposed to be an arabinosyltransferase based on sequence homology with *M. smegmatis embA* (Cole *et al.*, 1998; Escuyer *et al.*, 2001), although there is no direct evidence for its biochemical activity *in vitro* or *in vivo*. We could not determine the biological consequences of *embA* deletion in *M. tuberculosis*, since we were unable to construct a deletion mutant. However, based on the premises that EmbA_Msm_ synthesizes or transfers a disaccharide Ara*β*(1→2)Ara*α*1→ to an internal 5-linked Ara*f* of the linear glycan Ara*β*(1→2)Ara*α*(1→5)Ara*α*(1→5)Ara*α*(1→5)Ara*α*1→, we were able to use an enzyme assay to identify the function of EmbA_Mtb_.

**Fig. 2. f2:**
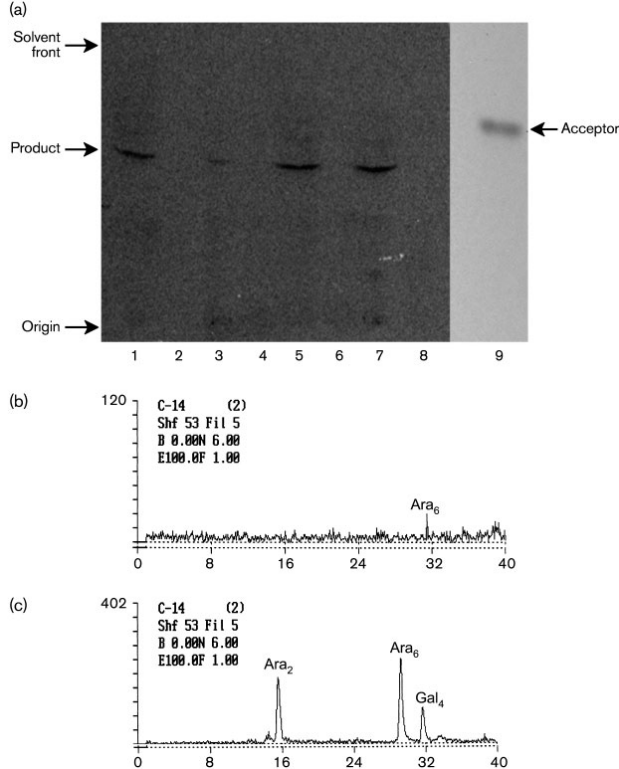
Arabinosyltransferase activity assays. Extracts from *M. smegmatis* strains were prepared and used in an *in vitro* assay for arabinosyltransferase activity. (a) TLC and (b) Dionex HPAEC was used to analyse the product formed after incubation with the pentasaccharide acceptor. (a) TLC of *M. smegmatis* strains. Wild-type (lanes 1 and 2), Δ*embA* (3 and 4), Δ*embA* complemented with *embA*_Msm_ (5 and 6) and Δ*embA* complemented with *embA*_Mtb_ (7 and 8). Lanes 1, 3, 5 and 7 are reactions with the addition of an acceptor pentasaccharide; lanes 2, 4, 6 and 8 are reactions with no acceptor. The acceptor pentasaccharide (lane 9) was visualized on TLC with *α*-naphthol. (b) The enzymically formed radioactive product was purified from the TLC plate and subjected to endoarabinanase digestion followed by Dionex HPAEC analysis, resulting in a single Ara_6_ peak. (c) ^14^C-labelled AG digested with endoarabinanase showing three peaks as Ara_2_, Ara_6_ and cyclic Gal_4_, as established by Xin *et al.* (1997).

Previous work has shown that an *M. smegmatis embA* mutant is unable to transfer a disaccharide to the pentasaccharide acceptor, but this function is restored by complementation with functional EmbA (Khasnobis *et al.*, 2006). We tested whether complementing this mutant strain with the *embA*_Mtb_ gene would restore this activity. As a control, we also complemented with the *embA*_Msm_ gene. Enzyme preparations from the *M. smegmatis* strains were used in a cell-free assay to detect arabinosyltransferase activity. TLC analysis of the reaction products revealed a slower migrating component as compared to the starting acceptor in *M. smegmatis embA* strains complemented with either *M. smegmatis* or *M. tuberculosis* alleles. The product in both strains was indistinguishable (Fig. 2a[Fig f2]), suggesting that *embA*_Mtb_ encodes a functional arabinosyltransferase with similar activity to EmbA_Msm_.

The mobility of the radioactive product does not indicate the number of arabinosyl residues incorporated on the pentasaccharide acceptor or the nature of the product. We therefore subjected the purified radioactive product excised from TLC to endoarabinanase digestion followed by Dionex HPAEC. Oligosaccharides were eluted with a gradient of sodium acetate (0–1 M) in 10 % NaOH. The digested sample yielded a single peak with a retention time comparable to the retention time of the terminal hexasaccharide Ara_6_ (Fig. 2b[Fig f2]) released from the ^14^C-labelled AG (Fig. 2c[Fig f2]). This result confirmed the formation of a heptamer by incorporating two Ara*f* residues on the pentasaccharide acceptor and was in full agreement with the product formed and analysed for *M. smegmatis* (Khasnobis *et al.*, 2006). In the biosynthetic product, which is a heptamer, there is only one site amenable to the *Cellulomonas* endoarabinanase action yielding one labelled Ara_6_ (Khasnobis *et al.*, 2006) leaving behind one unlabelled Ara with the octyl aglycon. The retention time of Ara_6_ is close to cyclic Gal_4_, which will not be detected as this acquires no radioactivity in this assay.

### Neutral sugar and glycosyl linkage composition

Neutral sugar composition of AG from the Δ*embA*_Msm_ strain (1.2 : 1) showed diminution of the total Ara content compared to the *embA*_Mtb_-complemented strain (2.2 : 1) which was in accordance with that of wild-type *M. smegmatis* (Escuyer *et al.*, 2001). The sugar composition was calculated based on a single rhamnosyl (Rha) residue per AG chain. Δ*embA*_Msm_ had no significant effect on the arabinosylation of LAM.

Purified LAM and soluble AG from Δ*embA*_Msm_ and Δ*embA*_Msm_ complemented with *embA*_Mtb_ were per-*O*-methylated and hydrolysed, reduced, acetylated and analysed by GC/MS to determine glycosyl linkage compositions. Analysis of AG yielded a molar ratio of tAra*f* (8.7 %), 2-Ara*f* (13.4 %), 5-Ara*f* (56.3 %), 3,5-Ara*f* (21.5 %) for Δ*embA*_Msm_ and a molar ratio of tAra*f* (13.2 %), 2-Ara*f* (14.1 %), 5-Ara*f* (52.6 %), 3,5-Ara*f* (20 %) for Δ*embA*_Msm_ complemented with *embA*_Mtb_. Thus complementation of the mutant with *embA*_Mtb_ clearly resurrected the defect in tAra*f*, 2-Ara*f* and reduced 5-Ara*f* which would occur if more branching is introduced. These results are in agreement with the cell-free assay, which resulted in the branched heptamer. We did not observe any changes in the composition of LAM in both Δ*embA*_Msm_ and Δ*embA*_Msm_ complemented with *embA*_Mtb_.

### Identification of the promoter for *embA*

The genomic organization of *embA* is shown in Fig. 1. Previous work in *M. smegmatis* suggested that *embA* is expressed either from its own promoter (Escuyer *et al.*, 2001) or from a polycistronic message encompassing the *embCAB* region (Telenti *et al.*, 1997). However, the promoter(s) were never directly identified and no data are available for *M. tuberculosis*. Other data suggest that the EmbR regulatory protein binds to the upstream region of *embA*_Mtb_ and that *embA* is subject to regulation by EmbR in *M. smegmatis* (Sharma *et al.*, 2006), although again, no data are available for *M. tuberculosis*. Because these experiments have not always been conducted in the native host, we investigated expression of *embA*_Mtb_ in its native host and in *M. smegmatis*.

**Fig. 3. f3:**
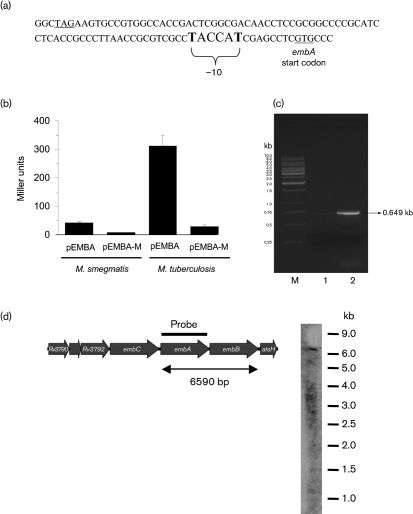
Promoter activity of the *embA* upstream region. (a) Sequence of the *embCA* intergenic region carrying P*_embA_*_(Mtb)_. The stop and start codons of *embC* and *embA* are underlined; the predicted −10 region is in large text. The bases in bold were mutated to Gs in plasmid pEMBA-M. (b) Promoter activity from P*_embA_*_(Mtb)_ in *M. smegmatis* and *M. tuberculosis*. *M. smegmatis* transformants were grown in 10 ml 7H9-OADC for 30 h. *M. tuberculosis* transformants were grown in 10 ml stationary cultures of 7H9-OADC-Tw for 14 days. Results are the means±sd of three individual transformants each assayed in duplicate and are expressed as Miller units. (c) RT-PCR analysis. Agarose gel analysis of RT-PCR on the *embC*–*embA* and *embA*–*embB* junctions of the *embCAB* locus. PCR with primers EMBCF2 and EMBAR failed to produce the expected 0.795 kb product (lane 1) while the primer pair EMBAF1 and EMBBR produced the expected 0.649 kb product (lane 2). Lane M is a 1 kb DNA ladder. (d) Genetic organization of the *emb* region on the *M. tuberculosis* chromosome and Northern analysis of total RNA from *M. tuberculosis* hybridized to labelled *embA*.

To determine if a functional promoter for *embA* exists immediately upstream of the gene, we cloned the *embC* and *embA* intergenic region (Fig. 1) into the promoter-probe vector pSM128 (Dussurget *et al.*, 1999), an integrating vector (which is therefore present in only one copy). We assayed promoter activity of this region in *M. smegmatis* and *M. tuberculosis* (Fig. 3). A functional promoter was present in this region as assayed by *β*-galactosidase activity. Interestingly, the activity of the promoter was significantly different in the two species (*P*<0.05 using Student's *t*-test), with a sevenfold higher expression level in its native host (*M. tuberculosis*). These results indicate that activity of the *embA*_Mtb_ promoter is not equivalent in the two organisms.

A predicted −10 promoter sequence is found upstream of *embA*_Mtb_ (Fig. 3a[Fig f3]). We confirmed that this was a functional promoter region using site-directed mutagenesis. Changing the sequence TACCAT to GACCAG (pEMBA-M) resulted in abolition of promoter activity, indicating that this is a functional promoter region (Fig. 3).

### *embA* is co-transcribed with *embB* in *M. tuberculosis*

Our data showed that *embA* has its own promoter and should be independent of *embC*. However, this did not reveal whether *embA* and *embB* are co-expressed. We used RT-PCR and Northern blotting to determine if *embA* was co-transcribed with *embC* and *embB*. We first utilized RT-PCR to determine if the genes were co-expressed. Primer pair EMBAF1 and EMBBR spanning the *embA*–*embB* junction was used; a 649 bp product was obtained, indicating that a co-transcript is present. In contrast, primer pair, EMBCF2 and EMBAR covering the *embC*–*embA* junction did not give rise to a product, indicating a lack of co-transcription (Fig. 3c[Fig f3]). This result was confirmed by Northern-blot analysis (Fig. 3d[Fig f3]). A single transcript of approximately 6.6 kb was detected using *embA*, corresponding to the length of both *embA* and *embB* (6590 bp). Thus our results demonstrate that *embA* is transcribed independently of *embC* but is co-transcribed with *embB*.

### Expression of P*_embA_*_(Mtb)_ during different growth phases

AG is a key component of the mycobacterial cell wall and it is possible that different amounts may be required during different growth phases, depending on the amount of cell wall being newly synthesized. For example, during active growth one would expect an increased requirement for new cell wall biosynthesis. Similarly, biosynthesis of AG could be reduced during stationary phase or in other non-replicating conditions where cell division has ceased. In contrast, the confirmation that *embA* is essential suggests that constitutive expression should be required for cell survival.

**Fig. 4. f4:**
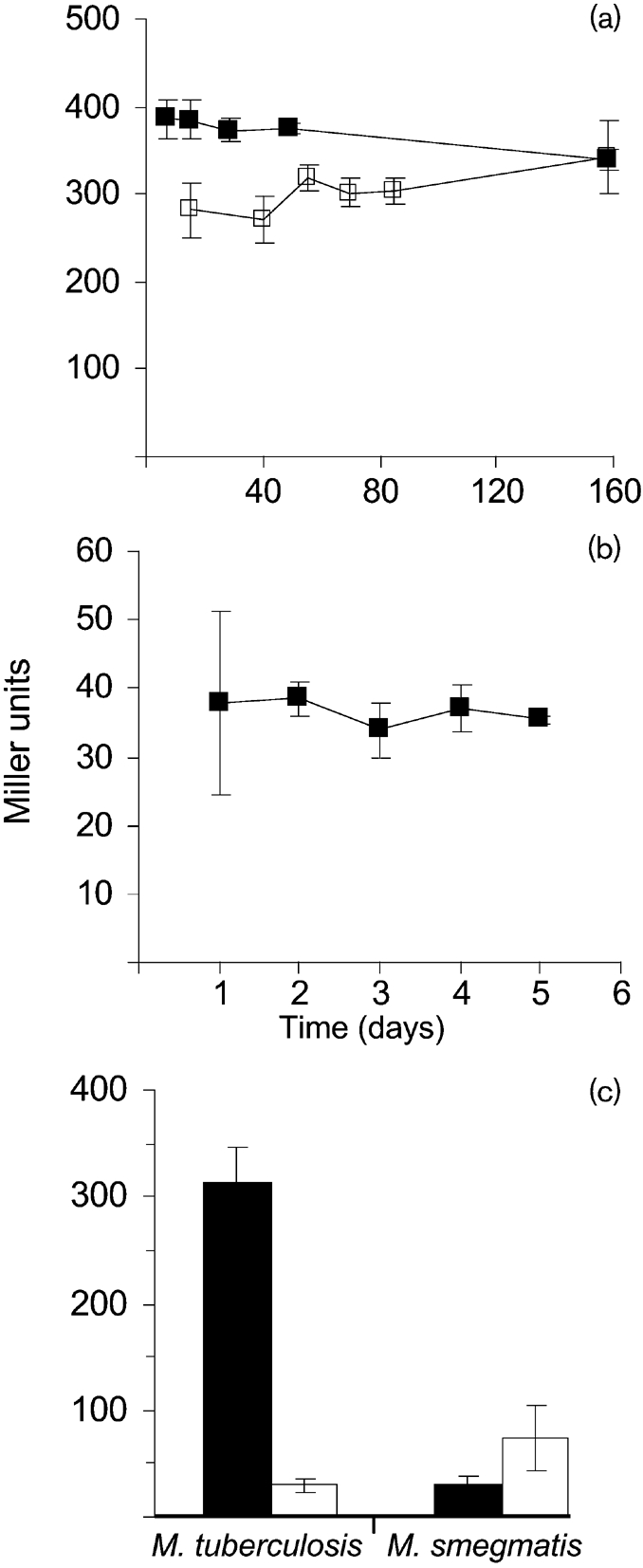
P*_embA_*_(Mtb)_ activity during aerobic growth and under hypoxic conditions. (a) Aerobic growth. *M. tuberculosis* was grown in 10 ml static cultures of 7H9-OADC-Tw (open symbols) or on 7H10-OADC plates (filled symbols). (b) *M. smegmatis* was grown in 10 ml 7H9-OADC. (c) Hypoxic growth. P*_embA_*_(Mtb)_ activity in hypoxic conditions (white columns) is compared to aerobic conditions (black columns). *M. tuberculosis* was grown under hypoxic conditions for 3 weeks and *M. smegmatis* for 2 weeks. Results are the means±sd of three individual transformants each assayed in duplicate and are expressed as Miller units.

We looked at P*_embA_*_(Mtb)_ activity during growth and under conditions of non-replicating persistence. Promoter activity was measured in *M. smegmatis* and *M. tuberculosis* to determine if there were any further differences (Fig. 4). Promoter activity was measured over 158 days in liquid and on solid media for *M. tuberculosis*. P*_embA_*_(Mtb)_ was more active in the cells grown on solid medium compared to liquid medium. In both media, no significant variation in activity was observed at different growth phases, indicating that P*_embA_*_(Mtb)_ is expressed to a constant level during growth and is not switched off during stationary phase (Fig. 4a[Fig f4]). In contrast, P*_embA_*_(Mtb)_ activity was markedly downregulated (tenfold) during a hypoxically induced non-replicating state (Fig. 4c[Fig f4]). A similar pattern of expression was seen during growth in *M. smegmatis*, with constitutive expression throughout the growth cycle, but at a lower level (Fig. 4b[Fig f4]). In contrast, a twofold upregulation of P*_embA_*_(Mtb)_ was seen under hypoxic conditions (*P*=0.02) (Fig. 4c[Fig f4]).

### Stress and drug treatments do not induce P*_embA_*_(Mtb)_

P*_embA_*_(Mtb)_ activity was assayed in response to oxidative stress generated by hydrogen peroxide exposure in *M. tuberculosis*. For these experiments, catalase and albumin were omitted from the media. No change in promoter activity was seen (data not shown). The experiment was also conducted in *M. smegmatis*, where no significant changes were seen in response to 5, 10 or 15 mM hydrogen peroxide exposure (data not shown).

Previous work has suggested that both *embC* and *embB* are upregulated in response to ethambutol treatment, although *embA* was not measured (Papavinasasundaram *et al.*, 2005). We looked at *embA* promoter activity after ethambutol or ofloxacin treatment in *M. tuberculosis* (data not shown). Antibiotics were added at 0.5× MIC. No induction of promoter activity was seen in response to ethambutol or ofloxacin treatment, demonstrating that expression of *embA* is not controlled in response to ethambutol exposure. Similarly, no induction of *embA* was seen in *M. smegmatis* (data not shown).

## DISCUSSION

### Essentiality of *embA* in *M. tuberculosis*

*embA* is a non-essential gene in *M. smegmatis*, although *embA* knockouts show an altered morphology and a slower growth rate than the wild-type, and AG synthesis and cell-wall permeability are affected (Escuyer *et al.*, 2001). It is not immediately obvious why EmbA plays a more critical role in *M. tuberculosis*. One possibility is that EmbB in *M. smegmatis* is able to function independently of EmbA, so that deletion of one enzyme is possible. This hypothesis is supported by the fact that a double *embAB* mutant could not be constructed in *M. smegmatis* (unpublished data). In *M. tuberculosis*, it is clear that EmbB cannot complement the function of EmbA. We have also attempted to delete *embB* in *M. tuberculosis*, but have been unsuccessful, indicating that it may also be independently essential.

### Differential expression of P*_embA_*_(Mtb)_ in *M. smegmatis*

We have shown that *embA*_Mtb_ has its own promoter and so can be independently expressed from *embC* and possibly *embB*. The activity of the *M. tuberculosis* promoter was markedly different in *M. smegmatis*, indicating that the latter species is not a good genetic model for the study of *embA*. In particular, the overall level of expression of P*_embA_*_(Mtb)_ was sevenfold lower in *M. smegmatis*. Recent work has suggested that PknH, a serine threonine kinase, is responsible for the phosphorylation of EmbR, which in turn is required for positive control of the *embCAB* genes (Sharma *et al.*, 2006). *M. smegmatis* does not possess a homologue of PknH and this may explain partly why P*_embA_*_(Mtb)_ is underexpressed in this background. The differential activity of the promoter in the two species in response to hypoxia could also be attributed to the lack of PknH, although the hypoxic response of *M. smegmatis* is not exactly the same as *M. tuberculosis* (Wayne & Sohaskey, 2001).

In conclusion, we have demonstrated that even though the gene products for the *embA* arabinosyltransferase in *M. tuberculosis* and *M. smegmatis* are similar, there are significant differences between the physiological requirements. In addition, the *M. tuberculosis embA* promoter is constitutively active, but differentially expressed in *M. smegmatis*. Further work using conditional expression systems as developed recently (Blokpoel *et al.*, 2005; Carroll *et al.*, 2005; Ehrt *et al.*, 2005) could help to determine why this arabinosyltransferase is critical for the survival of tubercle bacilli.

## Figures and Tables

**Table 1. t1:** Gene switching to confirm essentiality *embA* del-int strains (Tame 91 and Tame 93) were electroporated with control vector (*oriM*, pAGAN40) or switching vector (pUC-Hyg-Int) and transformants selected on hygromycin (H) or hygromycin and gentamicin (HG). Transformation efficiencies per μg input plasmid DNA are given. nd, Not determined.

**Strain**	**pAGAN40**		**pUC-Hyg-Int**	
	**H**	**HG**	**H**	**HG**
Tame 91	1.85E+08	2.18E+08	3.20E+05	2.15E+05
Tame 93	1.00E+07	nd	1.00E+05	nd

## Abbreviations

- AG, arabinogalactan
- DCO, SCO, double, single cross-over
- del-int, one deleted copy and one integrated copy of *embA*
- HPAEC, high-pH anion-exchange chromatography
- LAM, lipoarabinomannan
- mAGP, mycolyl-AG-peptidoglycan (complex)
- OADC, oleic acid, albumin, dextrose, catalase
